# Analysis of the Structure of Heavy Ion Irradiated LaFeO_3_ Using Grazing Angle X-ray Absorption Spectroscopy

**DOI:** 10.1021/acs.inorgchem.3c01191

**Published:** 2024-05-02

**Authors:** Luke T. Townsend, Claire L. Corkhill, David R. Hewitt, Amy S. Gandy, Neil C. Hyatt, Martin C. Stennett

**Affiliations:** † NucleUS Immobilisation Science Laboratory, Department of Materials Science and Engineering, The University of Sheffield, Sheffield S13 JD, U.K.; ‡ School of Earth Sciences, University of Bristol, Bristol BS8 1RJ, U.K.; § School of Mechanical and Materials Engineering, Washington State University, Pullman, Washington 99164, United States

## Abstract

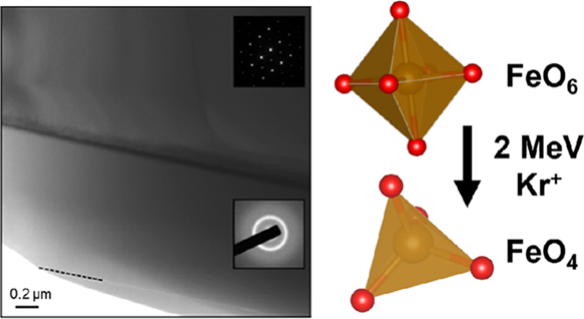

Crystalline ceramics are candidate materials for the immobilization of radionuclides, particularly transuranics (such as U, Pu, and Am), arising from the nuclear fuel cycle. Due to the α-decay of transuranics and the associated recoil of the parent nucleus, crystalline materials may eventually be rendered amorphous through changes to the crystal lattice caused by these recoil events. Previous work has shown irradiation of titanate-based ceramics to change the local cation environment significantly, particularly in the case of Ti which was shown to change from 6- to 5-fold coordination. Here, this work expands the Ti-based study to investigate the behavior in Fe-based materials, using LaFeO_3_ as an example material. Irradiation was simulated by heavy ion implantation of the bulk LaFeO_3_ ceramic, with the resulting amorphous layer characterized with grazing angle X-ray absorption spectroscopy (GA-XAS). Insights into the Fe speciation changes exhibited by the amorphized surface layer were provided through quantitative analysis, including pre-edge analysis, and modeling of the extended X-ray absorption fine structure (EXAFS), of the GA-XAS data.

## Abstract 2

LaFeO_3_ was exposed to 2 MeV Kr^+^ ions to observe the structural changes that take place upon irradiation. Using a combination of analytical techniques, including TEM and GA-XAS, it was shown that the coordination of Fe changed from 6-fold in the pristine material- to 4-fold in the irradiated material. These findings present an important step toward understanding the behavior of potential radioactive wasteform materials under irradiating conditions.

## Introduction

The safe disposal of actinide materials requires a predictive model of wasteform evolution, within the context of the geological disposal concept, based on a mechanistic understanding of the effect of radiation damage on the structure and dissolution behavior of the host material. Development of such an understanding must draw on a combination of systematic experimental approaches, including: the study of metamict minerals, doping with short-lived transuranic species, irradiation by fast neutrons, and irradiation by energetic ions.^1−9^ Of the processes that take place in actinide-containing wasteforms, the accumulation of radiation damage arising from α-decay of actinide species is a key concern for the long-term stability and performance of ceramic actinide hosts. The consequence of radiation-induced amorphization may be volume expansion of the ceramic material, sufficient to cause macroscopic cracking and hence an increase in the surface area available for dissolution. Such changes to the ceramic wasteform may impact the underpinning safety case for geological disposal and therefore need to be understood at a fundamental mechanistic level. The focus of this study is to understand the effect of ex situ ion beam irradiation on cation speciation in candidate ceramics, specifically Fe in LaFeO_3_, for actinide immobilization using X-ray absorption spectroscopy (XAS). LaFeO_3_ was chosen as a candidate wasteform to explore, due to the ability of the La^3+^-containing A-site having the capacity to accommodate transuranic species, and the perovskite structure showing good radiation tolerance. The choice of Fe^3+^ as the B-site cation builds on previous work that explored Ti coordination geometry changes upon irradiation (from predominantly 6- to predominantly 5-fold coordination with respect to oxygen ligands),^10^ with an interest in investigating how a more redox-active transition metal may behave in these radiation damaged systems. Ion beam irradiation with heavy ions simulates the damage accumulated by the recoil of actinide elements undergoing α-decay. XAS is a particularly useful probe of the structure of aperiodic materials, providing information on the local structure around an absorber atom (i.e., number, distance, and type of coordinating atoms).^11,12^ Here, this work overcomes the limitations of previous investigations^13^ and utilizes a previously established methodology^10^ whereby X-ray absorption spectroscopy in grazing angle geometry is applied to probe only the amorphized surface layer of 2 MeV Kr^+^-irradiated LaFeO_3_, in isolation of the undamaged substrate.

## Results and Discussion

**Figure 1:**
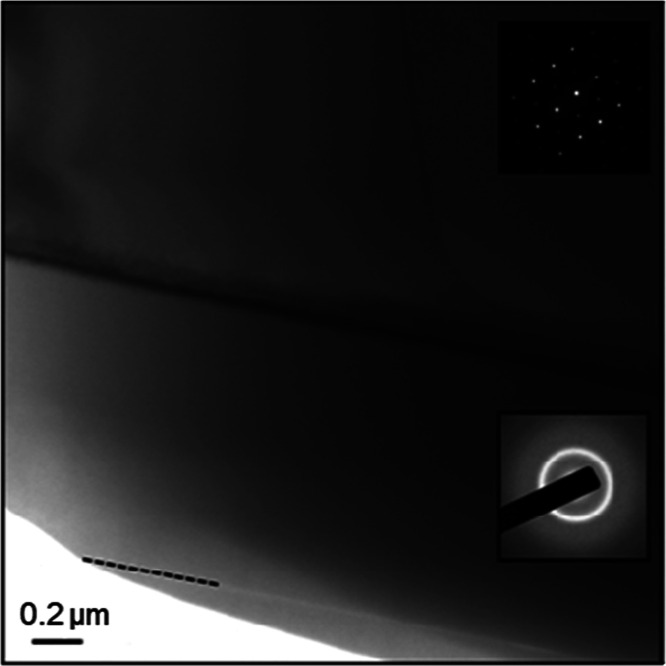
Transmission electron micrograph (TEM) and the corresponding electron diffraction (ED) patterns acquired from the two regions in the image of the irradiated sample.

Powder X-ray diffraction (PXRD) confirmed the synthesis of single-phase LaFeO_3_. All reflections could be indexed based on a *Pnma* (No. 62) cell with unit cell dimensions *a* = 5.5647(1) Å, *b* = 7.8551(1) Å, *c* = 5.5560(1) Å.^14^Supporting Information Figure S1 shows the XRD patterns for the pristine and irradiated samples with the major reflections indexed. The presence of diffuse intensity between 25 and 35° 2θ is consistent with the presence of amorphous material. The presence of an amorphous surface layer was confirmed by cross-sectional transmission electron microscopy (TEM) analysis. Figure 1 shows the transmission electron microscope image and corresponding electron diffraction (ED) patterns acquired from the implanted surface layer (shown in the lower inset) and the underlying pristine substrate (shown in the upper insert). The ED pattern from the implanted layer shows diffuse rings, characteristic of amorphous material, and an absence of discrete diffraction spots is clearly seen in the ED pattern acquired from the pristine substrate. The width of the amorphous layer is approximately 900 nm.

The ion beam radiation-damaged surface layer thickness was optimized in order to provide a maximum interaction volume for the study by XAS. This was achieved through the calculation of displacement profiles for LaFeO_3_ (Supporting Information Figure S2) using the program SRIM.^15^ Using parameters of 2 MeV Kr^+^ ions and assuming a common displacement energy of 50 eV, the SRIM calculation gives a damage profile with a peak at 600 nm. In general for nuclear ceramics, the amorphization dose (given in displacements per atom (dpa)) is considered to be independent of both the damage mode (whether α-decay or heavy ion beam irradiation) and the dose rate of heavy ions.^16^ Smith et al. reported that the amorphization dose for CaTiO_3_ perovskite (18 × 10^14^ ions cm^–2^) was approximately 3–5 times greater than that of zirconolite (3.5–6.1 × 10^14^ ions cm^–2^) when irradiated with 1.5 MeV Kr^+^ ions.^17^ Reported critical amorphization doses in other perovskites irradiated with 1.5 MeV Kr^+^ ions range between 2.5 × 10^14^ ions cm^–2^ for La_0.67_TiO_3_ and 10 × 10^14^ ions cm^–2^ for SrTiO_3_.^16−19^ Electron microscopy has indicated that the amorphous layer is approximately 900 nm thick in our sample, which suggests (based on SRIM calculations) a critical amorphization dose of approximately 5 dpa. Won et al. reported the critical amorphization dose in SrTiO_3_ to be approximately 4 dpa.^20^

**Figure 2:**
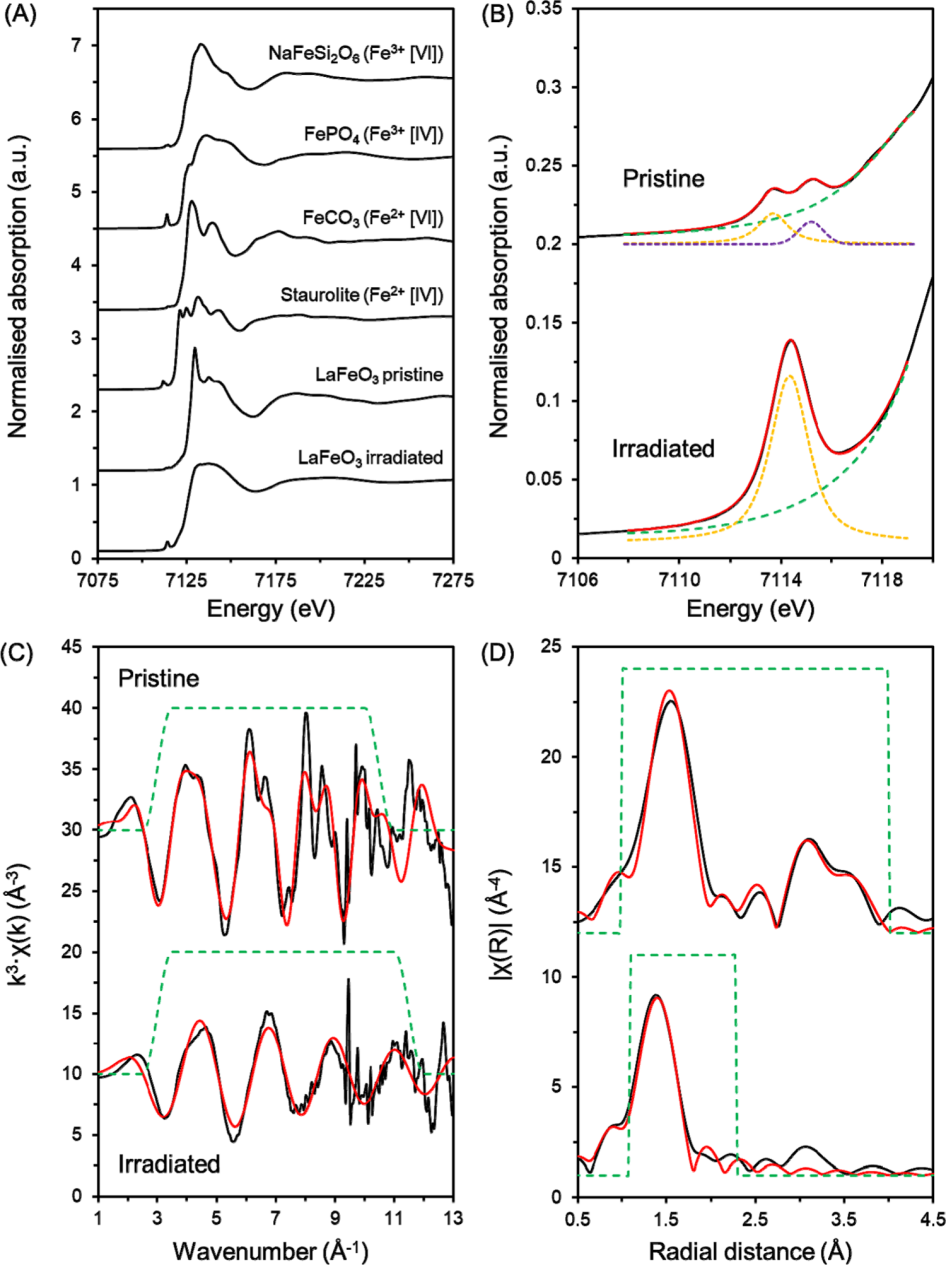
Fe K-edge X-ray absorption spectroscopy data of the pristine and irradiated LaFeO_3_ samples. (A) XANES spectra of the LaFeO_3_ samples and the corresponding standards (with Fe charge and local O coordination number (given in squared bracketed Roman numerals)); (B) pre-edge peak fitting where black lines are data, red lines are best fits, green dashed lines are the fit baseline, and yellow and purple dashed lines are pseudo-voigt peaks used in the fitting process; (C) *k*^3^-weighted EXAFS; (D) Fourier transform of the *k*^3^-weighted EXAFS.

**Figure 3:**
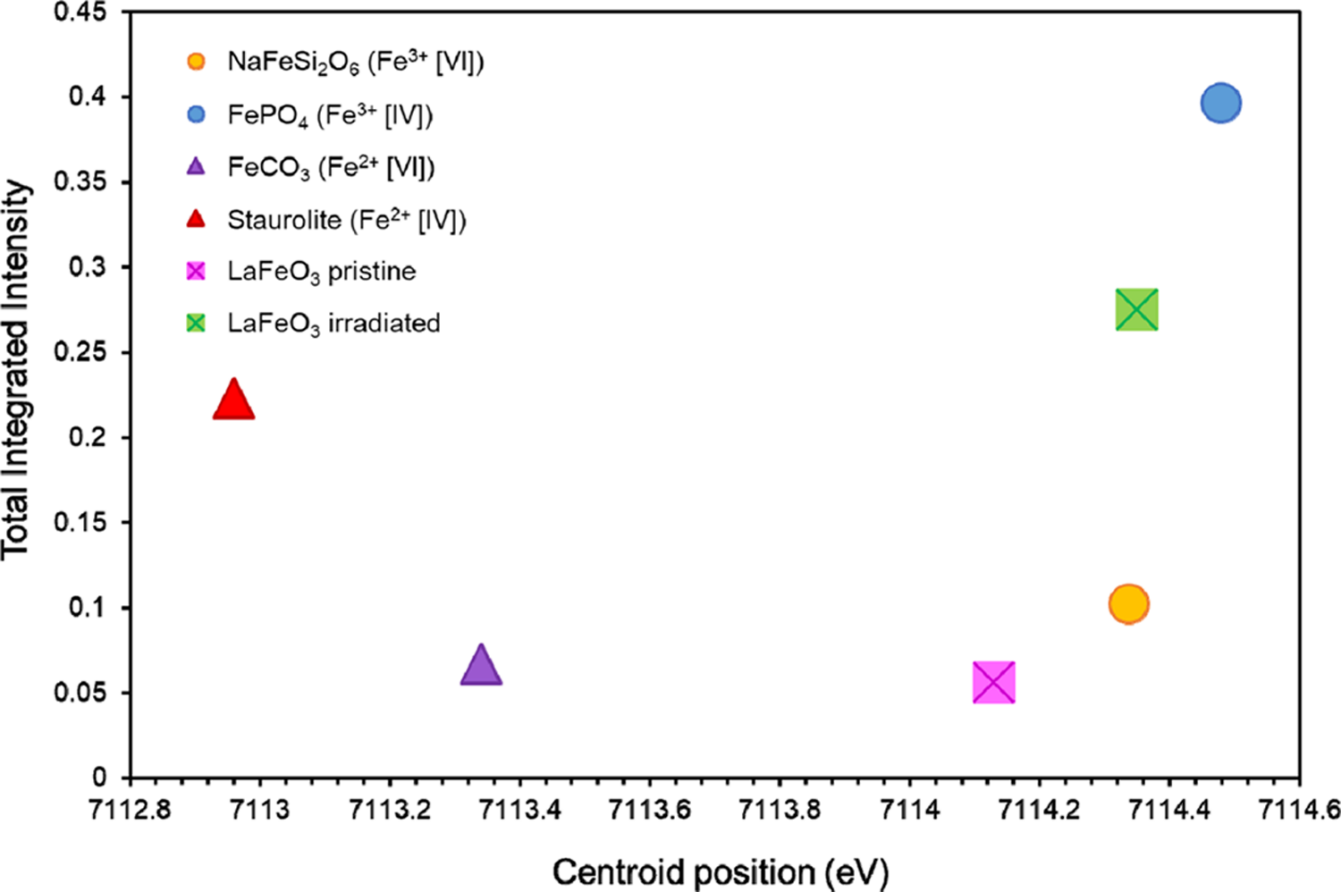
Results of the Fe K-edge pre-edge peak fitting presented as centroid position (eV) vs total integrated intensity (a.u.) of the fitted pre-edge peaks.

Using the SRIM calculations, angles were calculated to perform GA-XAS (Fe K-edge) at a depth that probed the amorphized layer at the surface of the irradiated LaFeO_3_ (with the same experiment being performed on the pristine LaFeO_3_ counterpart for comparison). Upon qualitative analysis of the X-ray absorption near-edge structure (XANES), neither the pristine nor irradiated LaFeO_3_ samples compared well with the suite of Fe standards indicating that the Fe in the LaFeO_3_ was in a unique coordination environment, both pre- and post-irradiation (Figure 2A). However, a clear change in Fe speciation can be observed with the broad relatively featureless XANES region of the irradiated LaFeO_3_ replacing the sharp, well-defined features in the XANES of the pristine sample (Figure 2A). The featureless nature of the irradiated sample indicated a significant loss of medium- to long-range order in the sample, corroborating the data obtained from the TEM and XRD. While the XANES region did not provide much insight into the specifics of the Fe local environment, the pre-edge region of the XAS data has previously been shown^21,22^ to provide significant insight into both the Fe oxidation state and coordination environment (with respect to oxygen (O)) when fit with pseudo-voight peaks and compared to a suite of standards. Using the methodology established by Wilke et al.,^21^ pseudo-voigt peaks were fit to the pre-edge region of the pristine and irradiated LaFeO_3_ samples (Figure 2B) and the corresponding standards (Supporting Information Figure S3; NaFeSi_2_O_6_ (Fe^3+^, 6-fold coordination), FePO_4_ (Fe^3+^, 4-fold coordination), FeCO_3_ (Fe^2+^, 6-fold coordination), and Staurolite (Fe_2_Al_9_O_6_(SiO_4_)_4_(O,OH)_2_) (Fe^2+^, 4-fold coordination)). While the number of pre-edge peaks may be observed to change from two to one, this alone is not sufficient to confirm a formal coordination change as pre-edge regions are affected by multiple factors beyond simply coordination number. From the fitting process, a total integrated intensity of the peaks and a centroid position was obtained for each sample and standard and was plotted to infer changes in the speciation of the Fe in the pristine and irradiated LaFeO_3_ samples (Figure 3 and Table 2). Here, the pre-edge peak fitting indicated that while the Fe oxidation state remains constant (Fe^3+^), a change from 6-fold to 4-fold coordination, with respect to O, takes place upon irradiation. The changes in transition metal coordination environment upon irradiation have been observed for Ti (from majority 6-fold to majority 5-fold with respect to O) in other nuclear wasteform materials^10^, and as such this indicates the capability of Fe to accommodate radiation damage through a similar mechanism.

The local Fe coordination environment was further investigated through the fitting of the Fe K-edge extended X-ray absorption fine structure (EXAFS) data (Figure 2C, D and Table 1). Here, the pristine sample was modeled well to the expected structure of LaFeO_3_, whereby Fe is coordinated by 6 O at 1.98(1) Å, 8 La and 6 Fe backscatterers at 3.38(1) and 3.98(2) Å, respectively. Multiple scattering contributions were present from the O and Fe backscatterers, indicating a good degree of medium- to long-range order. Following irradiation, the LaFeO_3_ showed a clear loss of ordering, as seen qualitatively by the loss of peaks in the Fourier transform at ∼3–3.5 Å, and more quantitatively by the best fit of the EXAFS only encompassing the first O shell (Table 1). The best fit obtained for the irradiated LaFeO_3_ only required 4 O backscatterers at 1.87(6) Å. However, a *C*^3^ parameter was required to fit the shell indicating a slight distortion in the FeO_4_ tetrahedra. The reduction in Fe–O distance from 1.98(1) to 1.87(6) Å is consistent with the formation of an Fe(III)O_4_ tetrahedral local coordination environment (Fe–O interatomic distance in Fe(III)PO_4_ = 1.86 Å)^23^. The reduction in degeneracy of the first O path (from six to four), the shortening of the Fe–O bonds, and the associated distortion of the resulting FeO_4_ tetrahedra all indicate clear structural changes in the Fe environment upon irradiation. Such findings are concordant with the aforementioned XRD and TEM data while also directly corroborating analysis (pre-edge fitting) performed on the XANES.

**Table 1:** Fitting Parameters for the Fe K-Edge EXAFS Data Shown in Figure 2^a^

path										
	*E*_0_	parameter	O1	La1	Fe1	O1MS1	O1MS2	O1 Fe1MS1	*R*-factor	BVS
pristine	–3.4(8)	*N*	6	8	6	12	12	12	0.0184	3.27
		σ^2^ (Å^2^)	0.003(1)	0.012(2)	0.015(3)	0.007	0.007	0.019		
		*R* (Å)	1.98(1)	3.38(1)	3.98(2)	3.35	3.38	3.97		
irradiated	–7.8(55)	*N*	4						0.0263	2.94
		σ^2^ (Å^2^)	0.004(1)							
		*R* (Å)	1.87(6)							
		*C*^3^ (Å^3^)	0.00024							

a Both fits utilized an amplitude reduction factor (*S*_0_^2^) of 0.8. *E*_0_ is the shift in the Fermi level; *N* is the degeneracy of the path; σ^2^ is the Debye–Waller factor; *R* is the interatomic distance; *C*^3^ is the third cumulant fitting parameter; *MS* are multiple scattering paths; *R*-factor is a measure of the goodness-of-fit; and BVS is the bond valence sum.

## Conclusions

Overall, this study has shown that LaFeO_3_ can accommodate significant structural alteration upon irradiation, with the local Fe coordination environment changing from 6-fold to 4-fold with respect to O coordination. Such structural changes have been evidenced with XRD and TEM, and upon performing GA-XAS experiments, the local- to medium-range speciation of Fe has been elucidated. The findings of this work help to underpin the fundamental chemistry associated with the irradiation of crystalline ceramics materials (specifically LaFeO_3_) which will inform future use of these materials in immobilizing α-emitting radionuclides, such as U, Pu, and Am.

**Table 2:** Results of the Pseudo-Voigt Fitting of the Fe K-Edge Pre-Edge Peaks (Figures 1, 3, and S3)^a^

sample	centroid position (eV)	total integrated intensity (a.u.)	reduced χ^2^
NaFeSi_2_O_6_ (Fe^3+^ [VI])	7114.34	0.102	1.3 × 10^–7^
FePO_4_ (Fe^3+^ [IV])	7114.48	0.396	3.2 × 10^–6^
FeCO_3_ (Fe^2+^ [VI])	7113.34	0.034	1.9 × 10^–7^
staurolite (Fe_2_Al_9_O_6_(SiO_4_)_4_(O,OH)_2_) (Fe^2+^ [IV])	7112.96	0.223	7.6 × 10^–7^
LaFeO_3_ pristine	7114.13	0.056	2.0 × 10^–7^
LaFeO_3_ irradiated	7114.35	0.275	4.1 × 10^–7^

a The centroid position is the center position of the sum of the peaks, and reduced χ^2^ is a measure of the goodness-of-fit.

## Methods

The LaFeO_3_ powder sample was synthesized from oxide (La_2_O_3_, Fe_2_O_3_) precursors using conventional solid-state sintering methods. The batch powder (20 g) was mixed with the carrier fluid isopropanol in a 45 mL sialon pot with sialon milling media, and planetary milled at 300 rpm for 5 min. The resulting powder slurry was then dried (∼100 °C for 16 h) before sieving the dry powder cake (250 μm mesh) and transferring to an alumina crucible for calcination in air (1250 °C for 16 h). After heat treatment, the phase purity of the sample was determined using transmission powder X-ray diffraction (XRD) on a STOE Stadi P instrument with Cu Kα radiation (λ = 1.5418 Å). The calcine was then milled (300 rpm for 5 min), and pellets (20 mm diameter, 30 mm height) were pressed using a hardened stainless-steel die and 60 MPa of uniaxial pressure. Following vacuum sealing in latex gloves, the green pellets were cold isostatically pressed at 200 MPa before being transferred onto stabilized zirconia setter plates for sintering (8 h at 1450 °C). Sectioning of the sintered pellets was performed to produce 1 mm thick cylindrical sections using a low-speed diamond saw, and these sections were then polished to an optical finish (0.25 μm). Finally, the polished sections were thermally annealed (1 h at 1300 °C) to reveal the grain boundaries and to relax out any surface stresses generated during the cutting process.

Samples were irradiated at room temperature with 2 MeV Kr to a fluence of 2 × 10^16^ Kr ions/cm^2^ at the Ion Beam Centre at Helmholtz-Zentrum Dresden-Rossendorf, Germany. A displacement profile was calculated using the software package SRIM,^15^ which predicted the peak in the damage profile to occur at ∼600 nm. This calculation is based upon an assumed displacement of 50 eV because no cation and anion displacement energies are available for this specific system. This assumption is in line with methodologies employed in previous studies.^24,25^ X-ray diffraction patterns were acquired from the surface of pristine and irradiated monoliths using Cu Kα radiation in Bragg–Brentano geometry with a Siemens D5000 diffractometer, operating at 40 kV and 30 mA, with a graphite diffracted beam monochromator. The cross-sectional transmission electron microscopy (xTEM) sample was produced by mechanical thinning, followed by ion milling using a Gatan Precision Ion Polishing System (PIPS), to achieve electron transparency.

The Fe K-edge XAS measurements were performed on the X23A2 beamline at the National Synchrotron Light Source (NSLS), Brookhaven National Laboratory (BNL). X23A2 consisted of an unfocused bending magnet beamline (4.9 to 32 keV) with an optics setup incorporating a fixed exit Golovchenko–Cowan designed Si(311) monochromator (resolution ±0.3 eV), and a single bounce flat Rh-coated harmonic rejection mirror. Energy calibration of the monochromator was performed by collecting Fe K-edge XAS spectra on a Fe foil, with the resulting edge position (defined as the first inflection in the derivative of the absorption edge) aligned to the known Fe edge position value of 7112 eV. Fluorescence measurements utilized a Vortex ME-4 silicon drift detector. Transmission measurements were collected using a finely ground specimen of pristine LaFeO_3_ dispersed in polyethylene glycol to achieve a thickness of one absorption length. Fluorescence measurements were collected on both the irradiated and unirradiated (pristine) samples, with each monolith exposed to the X-ray beam in such an orientation that the beam grazed the sample surface at a shallow angle. A bespoke stage was utilized to mount the samples and allow for the required tilt of the sample in relation to the plane of the incoming beam to be set within an accuracy of 0.1°. Grazing angles were selected to maintain a path length of at least three absorption lengths across a range of incident X-ray energies within the top 700 nm of the surface. This resulted in an incident angle of 1.4° when aiming for a penetration depth of ∼400 nm. All XAS (including EXAFS) data preprocessing (including self-absorption correction) and analysis was performed using the Demeter software package (Athena and Artemis).^26^ All pre-edge fitting was performed using Larch.^27^ Self-absorption correction is a key step for GA-XAS experiments due to the potential for pronounced distortion in intensities of both the XANES and (less so) the EXAFS data (see Supporting Information for further evidence and details). As such, transmission measurements of the pristine sample were used to self-absorb the fluorescence GA-XAS data, and the assumption of a similar composition was used to correct the irradiated GA-XAS data.

## Data Availability

The data that support these findings are available upon reasonable request to the authors.

## Supporting Information Available

XRD patterns of pristine and irradiated LaFeO_3_ (1); displacements profile for LaFeO_3_ (2); Fe K-edge pre-edge fitting of Fe^2+^ and Fe^3+^ standards (3); additional details on self-absorption correction procedure with comparison between unprocessed and processed data (4), and alternate (simplified) EXAFS fit for the irradiated LaFeO_3_ data (5) (PDF)
