# Supplementary material for: Analysis of the Structure of Heavy Ion Irradiated LaFeO3 Using Grazing Angle X-ray Absorption Spectroscopy
Source: Inorg Chem. 2024 May 2;63(19):8531–6. doi: 10.1021/acs.inorgchem.3c01191 (PMC11094784; doi:10.1021/acs.inorgchem.3c01191)
Supplement: Supplementary file 1 — ic3c01191_si_001.pdf [file ic3c01191_si_001.pdf]

Supplementary Information:

Analysis of the structure of heavy ion irradiated $LaFeO_3$ using grazing angle X-ray absorption spectroscopy

Luke T. Townsend[1], Claire L. Corkhill*[1,2], David R. Hewitt[1], Amy S. Gandy[1], Neil C. Hyatt[2,3], and Martin C. Stennett[1].

[1]NucleUS Immobilisation Science Laboratory, Department of Materials Science and Engineering, The University of Sheffield, Sheffield, S13 JD, United Kingdom.

[2]School of Earth Sciences, University of Bristol, Bristol, BS8 1RJ, United Kingdom

[3]School of Mechanical and Materials Engineering, Washington State University, Pullman, WA, 99164, USA

*Corresponding authors: c.corkhill@bristol.ac.uk

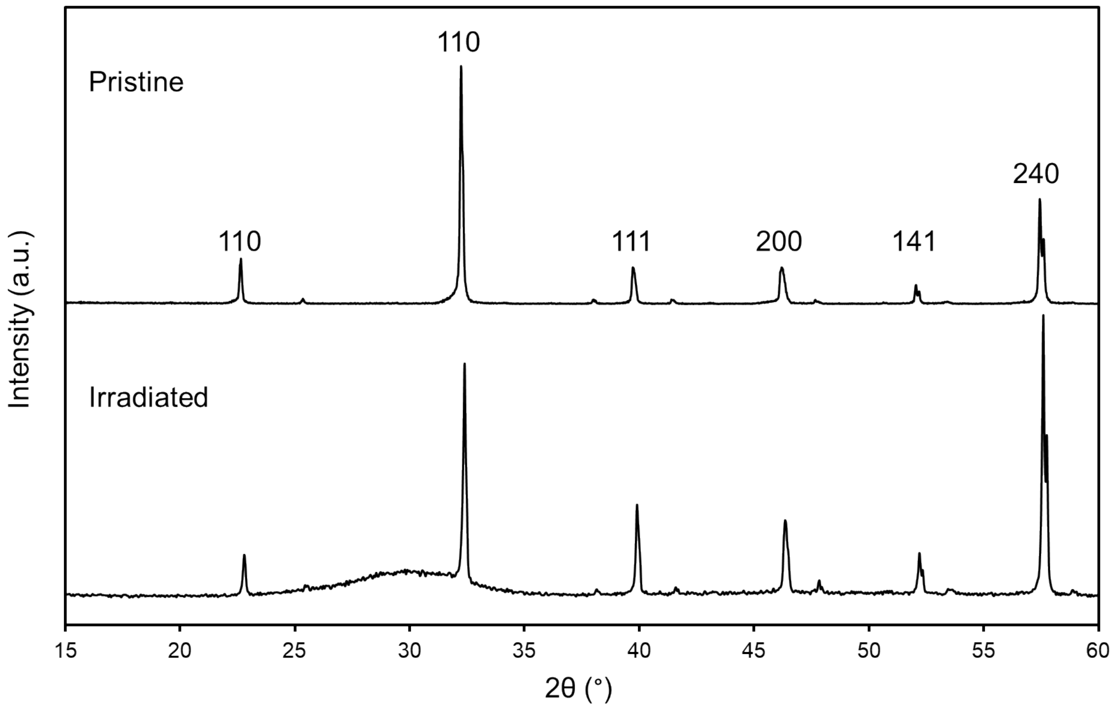

Figure S1. XRD patterns for pristine and irradiated $LaFeO_3$ samples. Indexing of major peaks based on [14].

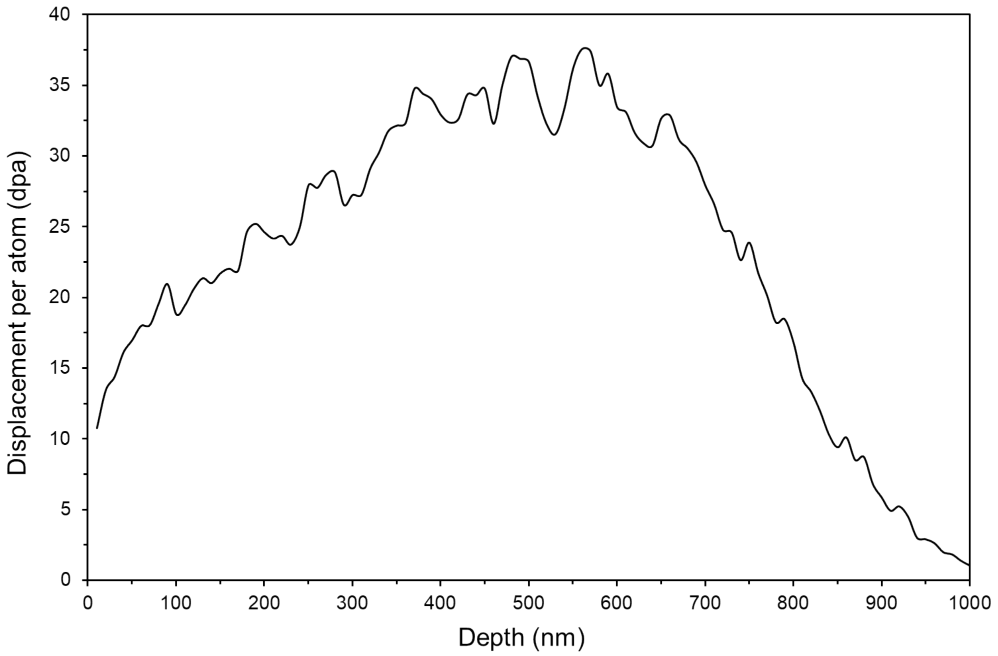

Figure S2. Displacement profile for $LaFeO_3$ irradiated with 2 MeV Kr^+ ions to a fluence of $2x10^{16}$ Kr ions/cm^2.

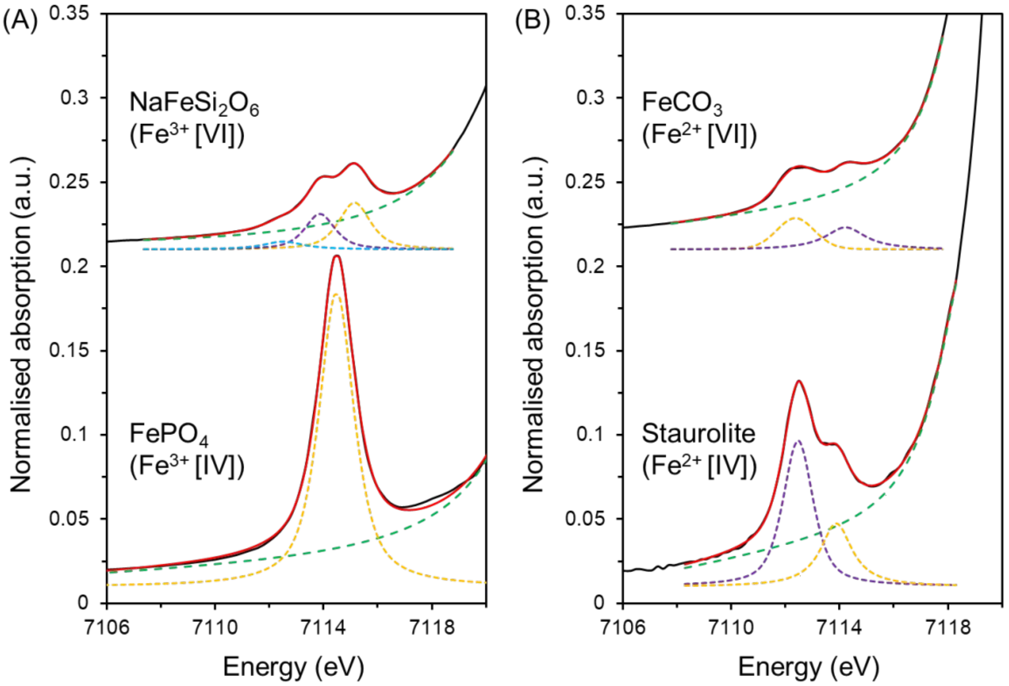

Figure S3. Fe K-edge pre-edge peak fitting of the (A) Fe^{3+} (top: $NaFeSi_2O_6$ (6-fold coordination); bottom: $FePO_4$ (4-fold coordination)) and (B) Fe^{2+} standards (top: $FeCO_3$ (6-fold coordination); bottom: Staurolite ($Fe_2Al_9O_6(SiO_4)_4(O,OH)_2$) (4-fold coordination)). Solid black and red lines are data and fit, respectively; dashed green lines are baselines; dashed yellow, purple, and blue lines are pseudovoight peaks used in the fitting process.

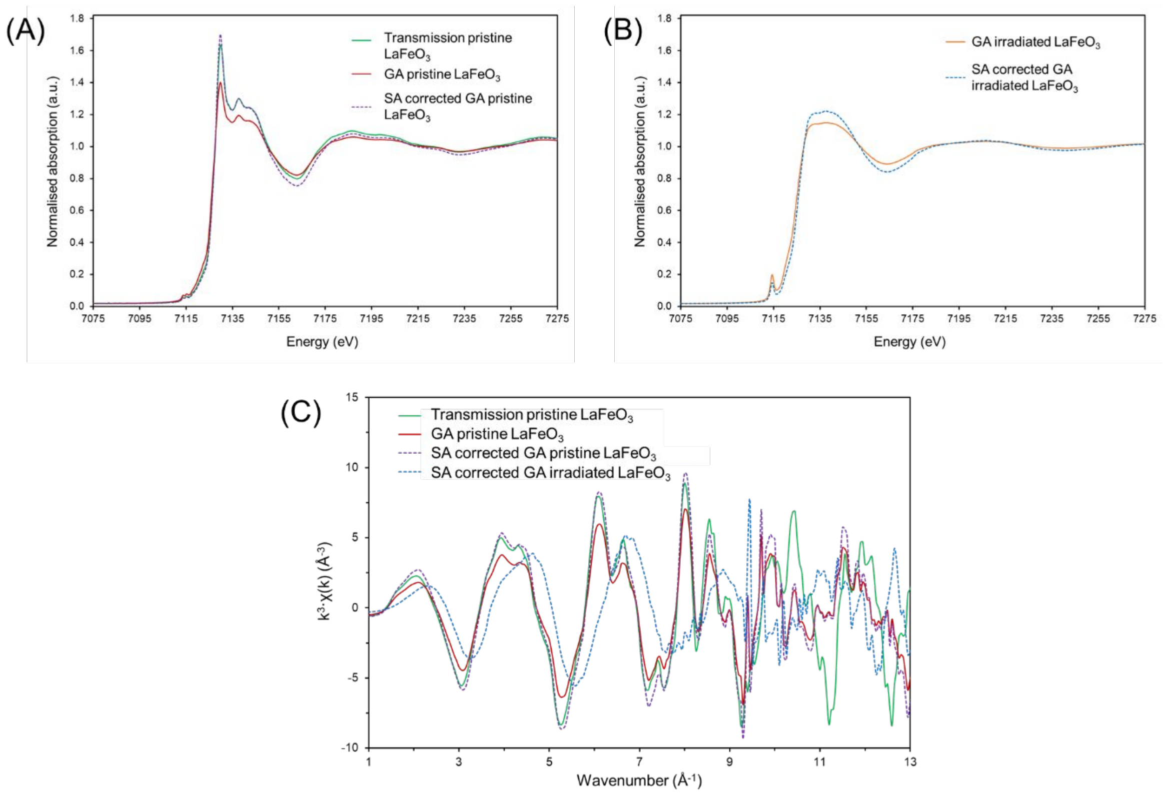

Figure S4. Comparison between the transmission, glancing-angle (GA), and self-absorption (SA) corrected XANES (A & B) and EXAFS (C) for the pristine and irradiated $LaFeO_3$ samples. The GA-XANES data for the pristine sample was self-absorption corrected to the pristine transmission data using the chemical formula for $LaFeO_3$ and the incident angle of the GA-XAS experiment (~1.4 °) before applying the same correction to the irradiated data. For EXAFS, a similar procedure was applied but utilising a Booth algorithm (with the approximate density of $LaFeO_3$ (6.40 g cm^{-3})). During the fitting of the EXAFS, S_0^2 was iterated upon to compensate for any further self-absorption corrections that may have been required with a final value of 0.8 used.

Table S1. Alternate (simplified) fit for the irradiated $LaFeO_3$ sample. In this fit, the degeneracy (N) was allowed to refine with S_0^2 = 0.8 (the same value used in the fit presented in the main manuscript).

	E_0	Parameter	O1	R-factor	BVS
		N	4.01(86)		
Irradiated	-8.9(35)	σ^2 (Å^2)	0.004(3)	0.0299	3.05
		R (Å)	1.86(2)		

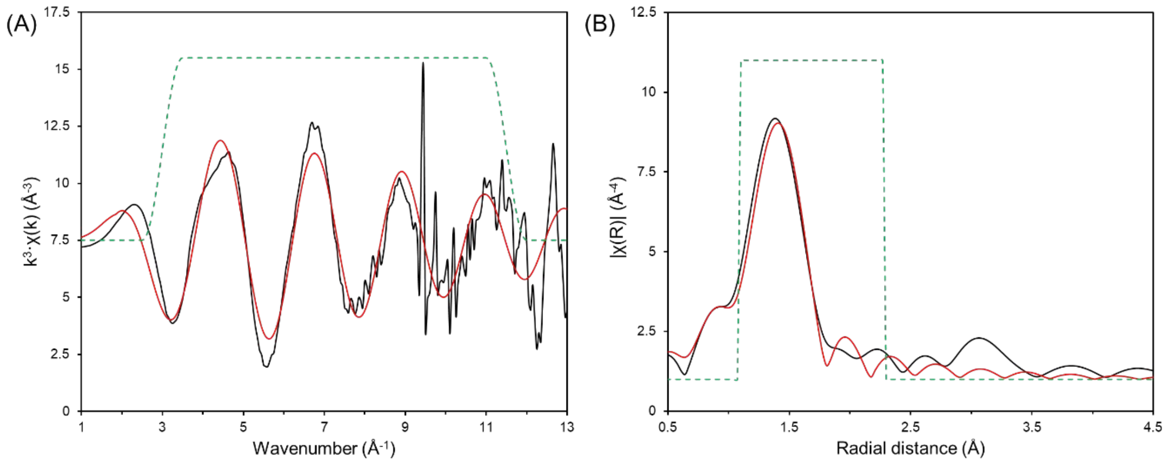

Figure S5. Alternate (simplified) EXAFS fit (red lines) for the irradiated $LaFeO_3$ sample data (black lines) presented as (A) k^3-weighted EXAFS and (B) the Fourier transform of k^3-weighted EXAFS. Fitting data is provided in Table S1.
